# Possibilities and Principles of Formation of Functionally Graded Ceramic Armor by Additive Manufacturing

**DOI:** 10.3390/ma18184370

**Published:** 2025-09-18

**Authors:** Eugene Medvedovski, Nahum Travitzky

**Affiliations:** 1 Independent Researcher, Ottawa, ON K2M 1E3, Canada; 2 Department of Materials Science, Friedrich-Alexander University of Erlangen-Nuernberg, 91058 Erlangen, Germany; nahum.travitzky@fau.de

**Keywords:** ceramic armor, functionally graded material, additive manufacturing, microstructure, ballistic fracturing

## Abstract

Ceramic armor with improved ballistic performance, lower weight, and, in specific cases, complex geometry is of high importance for the armor integrators and users. The combination of a high level of consolidation and the tailored heterogeneous structures of these ceramics may be important for reducing fracturing and delaying crack propagation under high-velocity ballistic impacts, therefore improving their ballistic performance. This can be attained by a beneficial combination of advanced ceramic compositions and processing, e.g., through an additive manufacturing (AM) approach. For example, the functionally graded (FG) monolithic ceramic armor obtained by AM can encompass a few layers, where the front layer has higher hardness, and the layer(s) behind may have higher fracture toughness. The ingredients reinforcing the ceramic structure may also be involved. The graded architecture should provide the internal stress reduction under high-velocity mechanical (ballistic) impacts. The reaction bonding that provides consolidation of the AM-based ceramic armor may also be involved. The considered principles of the FG materials’ formation may be employed for personnel, vehicular, and structural armor systems. The possibility of formation of ceramic armor with graded structures and compositions using AM was reviewed and analyzed for the first time. The proposed armor designs, which can be obtained by AM, may provide enhanced ballistic performance combined with lower weight.

## 1. Ceramic Armor and “Conventional” Manufacturing Methods

### 1.1. General Situation with Ceramic Armor Materials

Ceramic armor with improved ballistic performance, especially with multi-hit performance, lower weight, and, in particular cases, with relatively complex shapes and large surface areas is of high importance for armor integrators and users. Basically, ceramic armor systems consist of the hard monolithic ceramic or composite ceramic–metal armor body at the strike face and the soft elastic or ductile backing materials made of composite polymeric materials (e.g., aramid-based materials, like Kevlar^TM^, Twaron^TM^, or high-density polyethylene, like UHMWPE) or metals. In many cases, a spall protection sheet and the confinement layer are used to increase ballistic performance. Ballistic protection is defined by the armor system design and by the involved materials’ properties [1,2,3,4,5]. Shortly, upon ballistic impact (when the bullet velocity is greater than 700–800 m/s), the front hard ceramic body is cracked and broken, defeating the projectile with dissipating kinetic energy, and the residual energy is absorbed by the soft or ductile-reinforced backing material. This backing material also supports post-impact fracturing of the ceramic body, and it should capture the defeated projectile and fragments of the disintegrated ceramic body.

Ceramic armor materials should possess a combination of properties, such as high hardness (to be able to defeat a projectile), remarkable fracture toughness, high Young’s modulus and sonic velocity, high specific stiffness (defined as Young’s modulus/density), and optimal brittleness, which is defined by hardness, fracture toughness and Young’s modulus according to the formula *B = HV.E/K_Ic_^2^* (*HV* is Vickers hardness, *E* is Young’s modulus, and *K_Ic_* is fracture toughness) [3,4,6,7,8,9,10,11]. Thus, the ballistic energy dissipation ability may be expressed by the formulae: *D* = 0.36(*HV.c.E/K_Ic_*^2^) [8] or *D* = 0.36(*B.c*) [3,12,13]. Since ballistic energy dissipation also depends on ceramics’ phase composition and structure, the *D*-factor may be expressed as *D* = *B.c.S* (where *S* is a “structural” factor) [12,13], demonstrating that only a combination of physical properties and structure of armor ceramics should be considered in their evaluation and selection. Crack propagation, which is mostly defined by toughness, Young’s modulus, and sonic velocity, as well as structural features, at high-energy ballistic impacts, is very important in the materials disintegration and, hence, in the ballistic performance of ceramics [3,4,8,9,10,11]. Because of this, the modification (or “management”) of crack propagation, focusing on its delaying, could have a crucial role in the creation of ceramics with high ballistic performance. Ceramic structure also affects the speed of propagation of a shock wave occurring at the ballistic impact. Processing features also strongly affect the structure and homogeneity, residual stresses, and defects in the microstructure of ceramics, which, in turn, affect ballistic performance. Therefore, the ceramic armor can be improved through the appropriate material design combined with reliable technology, assisting in the delay of crack propagation.

Among different advanced ceramics, the most usable, at present, materials for armor applications are dense alumina, silicon carbide, and boron carbide ceramics or a combination thereof. Considering oxide materials, also alumina-zirconia (which is heavier than alumina) and alumina-mullite (which is lighter than alumina) ceramics, as well as spinel ceramics (specifically for transparent armor applications), demonstrated high ballistic performance. For non-oxide materials, lightweight Si_3_N_4_, SiAlON, AlN, and AlON can also be really good options, since they also demonstrated a high level of ballistic performance. Multi-phase ceramics made from this group can also be very efficient as armor materials. Ceramic–metal composites (e.g., B_4_C–Al, SiC–Al, Al_2_O_3_–Ti, and some others) should have high ballistic performance due to their enhanced toughness. The application of some hard borides and carbides (e.g., based on TiB_2_, WC, and TiC) as armor materials is seriously limited by their high weight. The mentioned ceramic or composite materials may have homogeneous small-grained structures or structures that are heterogeneous, especially designed structures. The latter materials may be (i) reinforced by platelets or whiskers, (ii) may be made with a specially selected particle size distribution, where a homogeneous matrix is reinforced by large grains, and (iii) may consist of structures obtained through a reaction-bonding mechanism or metal infiltration.

### 1.2. “Conventional” Manufacturing Routes of Ceramic Armor

**Figure 1:**
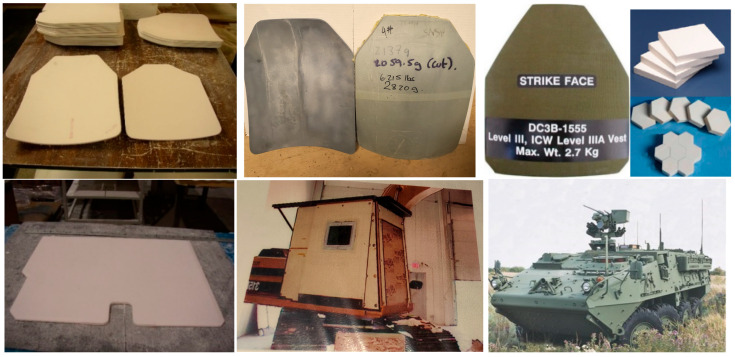
Body Armor Plates, Tiles and Panels Commercially Produced by “Conventional” Technologies (e.g., Slip Casting and Pressing).

Different processes are commonly employed for ceramic armor manufacturing, and the technology is selected based on the required size and shape of armor products (see Figure 1), quantity to be produced, available processing equipment, and experience of the manufacturer in certain processes. The processing methods include casting (slip casting or pressure casting into plaster or polymeric molds or gel casting), pressing, and injection molding to form the required shapes, followed by pressureless or pressure-assisted sintering. In all these methods, colloidal processing is a must because of the necessity of a reproducible structure with the required distribution of certain functional additives and minimal unpredictable flaws and defects, and because of a high level of compaction and sintering. Pressure-assisted consolidation technology, particularly, hot pressing as the most practical, is widely used for the carbide and boride armor ceramics. In this case, forming and sintering of the tiles or plates are often conducted from the starting powders without preliminary colloidal processing. In the cases of reaction-bonding or infiltration with a metallic ingredient, the porous ceramic preforms are shaped (by casting, pressing, etc.), and then the material consolidation is conducted at high temperatures and in selected gaseous atmospheric conditions through the bonding phase formation. Reaction-bonded silicon carbide (RBSC) or boron carbide (RBBC) ceramics, where reaction bonding occurs due to interaction of residual C with Si, are especially important and employed for ballistic armor [3,6,13,14,15,16]. Reaction-bonded ceramics can also be obtained through solidification of the molten ingredient, which can interact with the “base” ceramics providing additional bonding between the phases. It should be outlined that heterogeneous reaction-bonded SiC and B_4_C ceramics obtained through the infiltration process demonstrate good multi-hit ballistic performance compared to homogeneous ceramics composed of the same ingredients despite the latter have higher hardness and other mechanical properties. This is related to a reduced crack propagation under ballistic impacts of reaction-bonded ceramics [4,13]. Similar positive effect was also demonstrated for SiC–Al_2_O_3_ and SiC–Si_3_N_4_–Al_2_O_3_ armor ceramics, where the reaction bonding mechanism was realized through high temperature oxidation and interaction between the ingredients during pressureless sintering in an oxygen-rich atmosphere [4,13].

Manufacturing productivity depends on the selected materials and their associated fabrication technologies, including suitable production equipment. Thus, according to manufacturing experience, two or even three hundred body armor plates (single-, double-, or triple-curve) per day were successfully produced by slip casting technology from different alumina ceramics, or up to a hundred plates or more were produced from RBSC or RBBC by pressure casting or pressing of ceramic preforms and following infiltration with silicon. Simple armor tiles (alumina or SiC ceramics) with dimensions of 50–100 × 50–100 mm^2^ were produced with quantities of up to two—three thousand pieces per day using uniaxial pressing with only two press-die cavities (using more cavities at pressing will increase productivity accordingly). However, large complex shape alumina or RBSC ceramic flat or curved panels (with dimensions greater than 0.5 m length and width) used for vehicular or structural applications could be made by slip casting only with lower productivity, i.e., the components’ dimensions and shape complexity and related handling necessity also define productivity.

**Figure 2:**
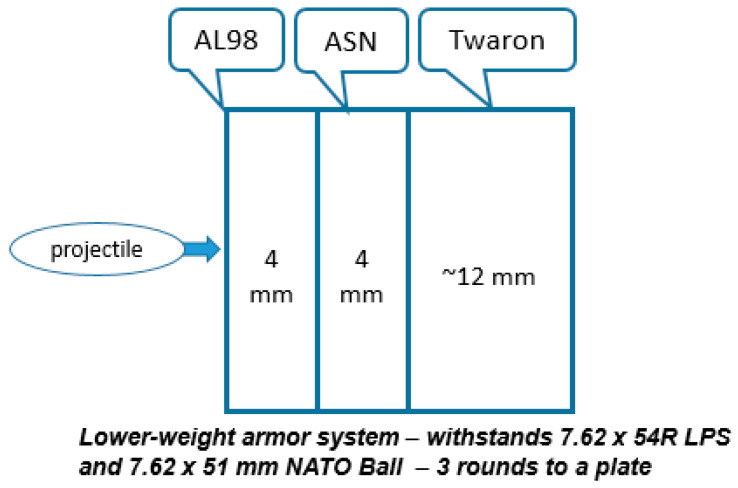
Graded Armor System Composed of Separately Produced Double-Curved Plates.

The briefly described methods allow producing monolithic ceramic components where the selected composition and structure are the same throughout the whole body. The component’s weight and performance (as well as properties) are defined by the nature of the ceramic materials, density, as well as by the structure, depending on the composition and processing features. The graded components’ architecture, consisting of the layers from different, separately fired materials, specifically “heavier” and “lighter” layers, may be more beneficial, providing the components’ weight reduction if the obtained composite’s performance remains on the required level. Thus, Li et al. [17] reported that the laminated Si_3_N_4_/BN can withstand ballistic impacts, but the penetration resistance of these composites was lower compared to monolithic Si_3_N_4_ ceramics. However, Yadav et al. [18] stated that laminated structures have improved ballistic performance over monolithic structures. Thus, the three-layer blocks made of AlN ceramic flat tiles bonded through the polyurethane adhesive outperformed the monolithic blocks of the same total thickness after ballistic testing. This is attributed to a reduced wave propagation velocity and crack arresting by polymer layers. Our earlier trials towards obtaining ceramic armor with a graded layered architecture were conducted to combine the benefits of different ceramics. Uniaxial pressing of several thin (1–2 mm thick) alumina ceramic sheets, made by tape casting, in one press-die and followed by firing, was not successful. The fired tiles had delamination and cracking related to binder burnout and, as a result, poor ballistic performance [4]. As well, lamination of the fired thin (1–2 mm) alumina tiles glued by adhesives did not provide adequate ballistic performance (tested with NATO Ball ammunition) compared to solid monolithic tiles; the front layers were strongly disintegrated under ballistic impact, and delamination was observed [4]. Only bonding of relatively thick, separately fired ceramic armor plates was rather effective; however, the manufacturing effectiveness was limited, and the productivity was not high because of the plates’ specific curvature. Thus, the manufacturing trials with thin alumina and SiC-based ceramic double-curve body armor plates (with ~4 mm thickness of each plate), bonded using special adhesives (see the schematics in Figure 2) achieved high multi-hit ballistic performance [4] and allowed reduction in the total weight of the armor (compared to monolithic alumina ceramics). However, it was difficult to reach a perfect match of the curvatures of two ceramic bodies after firing for accurate bonding, and this issue significantly reduced the manufacturing productivity and yield. This is especially notable for double- and, moreover, for triple-curved body armor plates. The formation of thinner ceramic layers (e.g., 2–3 mm) for higher versatility in design is even more difficult and less productive. Uniaxial pressing of ceramic powders with different compositions in one press-die, followed by a single firing cycle in order to create the graded structure, was not successful.

## 2. Potential for Functionally Graded Ceramics for Armor

Functionally graded (FG) ceramics, specifically with laminated architectures, may have high potential for armor applications by designing favorable combinations in lower weight, optimal structure and properties, and reduced crack propagation utilizing different materials [13,19]. The major principle of the functionally graded material (FGM) structure includes the creation of the continually varied microstructures and compositions to reach desirable mechanical and some other functional properties through the thickness of the whole component. The resulting graded structures allow the residual stress concentration reduction and the enhancement of bonding strength in laminates [20,21,22]. Basically, when stress waves created by a ballistic impact propagate through the interface of two dissimilar materials, a tensile force occurs at the stress wave transmission and reflection off the interface, which can cause the materials’ disbonding, especially in the case of a “strong” notable interface. This reduces the ability to absorb impact energy during the ballistic impact and reduces the armor system’s disintegration. One of the purposes and challenges of the FGM with reliable properties is to minimize the residual stress concentration, as well as enhance the bonding between the layers.

Numerous studies on ceramic or ceramic–metal FGM utilized pressure-assisted technology, e.g., hot pressing (HP) and spark plasma sintering (SPS), focused on consolidation of the processed bodies, as well as on minimizing the issues related to the shrinkage mismatch and the mechanical and thermal stresses between the layers with different compositions, which may occur at firing [20,23,24,25,26]. The materials may have either a rather smooth transition in the compositions or have a layered architecture, where the layers are “connected” by thin “transition” zones, specifically by placing the layers with high compressive stresses into the middle of the materials [27]. For example, ballistic testing of the FG ceramic armor (FGCA) based on TiB_2_–Ti with transitional compositions (up to 20 wt.-% of Ti) prepared through the powder processing and consolidated by SPS demonstrated promising results [28]. The most promising design was attributed to the composite’s structure where the hardest, TiB_2_–10Ti, layer was in the front, while the lower-hardness but tougher, TiB_2_–20Ti layer was behind. These authors indicated that the TiB_2_–10Ti composite predominantly failed by the intergranular mode of fracture, while the TiB_2_–20Ti composite predominantly failed by the transgranular mode, and the TiB_2_–10Ti/TiB_2_–20Ti FGM failed by the mixed mode of fracture with cleavage steps. The authors also indicated that an increase in the FGM thickness from 5 to ~8 mm led to degradation of ballistic performance (evaluated by measuring the depth of penetration to the backing). However, these results may be considered only as preliminary since the authors tested only small, 50 mm, discs with no proper bonding to an aluminum block placed behind the composite disc. Promising results in ballistic performance of FG cermet armor tiles made of SiC—Al with different ceramic/metal ratios and produced through powder metallurgy followed by hot pressing were also obtained by Aydin and Apalak [29] and Chouhan et al. [30]. Higher ballistic performance (a reduced depth of penetration) can be commonly attained for the composites designed by arrangement of the ceramic–rich zone with higher hardness in the strike face, placing the metallic-rich zones with higher toughness behind. This architecture provided better dissipation of the remaining energy of the projectile. However, the proposed designs and technology were suited mostly for the planar armor systems.

Another path of the FGM consolidation can be realized through metal infiltration, and this path has already been tested for armor ceramic composites. According to Wang et al. [31], porous B_4_C scaffolds prepared by gel casting, layer-by-layer (diameter ~105 mm with a total thickness of 20 mm), were infiltrated by aluminum under pressure, and the composites were preheated at 800 °C. Finally, the samples were sintered at 1900 °C in Ar. A smooth compositional transition allowed no layers’ delamination, which positively affected the composites’ ballistic performance (the composites with an aluminum backing were tested using the ammunition of a 7.62 × 53 mm AP with a hardened steel core). Aydin et al. [32] explored the influence of the thickness of laminated FG plates under ballistic impacts, varying the number of layers as 7, 12, 17, and 22, and these authors made an obvious conclusion that the layers’ thickness decreases the depth of penetration. The composite materials with rather thin coating layers and with significantly higher hardness (compared to the “major” material) or the materials, where the coating layer creates surface compressive stress, may also be considered.

In some studies, “conventional” forming technologies were explored for functionally graded ceramic armor (FGCA). Huang and Chen [33] ballistically tested small flat pressed tiles based on Al_2_O_3_-ZrO_2_ with varied ingredient contents. The recorded promising results were related to the delay in crack propagation under ballistic impact due to the graded composition in the ceramics. However, this compositional and technological approach would be rather inconvenient in industrial production, delaying productivity. Slip casting is another method that allows obtaining FGAC, using sequential casting of slips with different compositions in the same mold. Thus, Bermejo et al. [34] obtained FG-ceramics consisting of the Al_2_O_3_—5 vol.-% ZrO_2_ “thick” (~520 µm) layers separated by the Al_2_O_3_—30 vol.-% ZrO_2_ “thin” (~100 µm) layers with a total of nine layers (a total thickness of ~3 mm). High compressive stresses in the thin layers resulted in the edge crack formation in the layered composite. Bending testing showed stepwise fracturing compared to brittle failure for monolithic ceramics, i.e., a thin layer acts as a barrier for crack propagation. Our sequential casting trials included casting of alumina-zirconia slip to plaster molds first, then, after a certain time of dewatering, alumina slip was cast to the same mold, and finally alumina-mullite slip was cast. This procedure resulted in the formation of the five-layer body upon the casting process completion (casting was conducted to an actual “production” body armor plaster mold, providing a total thickness of ~9.5–10 mm of a green double- or triple-curved plate). Since the ceramic compositions involved in this process can be fired at the same temperature to reach complete densification and since these materials have almost the same shrinkage factors, the fired bodies did not have visible delamination and cracking. As expected, fired density (and weight) of this “three-composition” ceramics was lower compared to homogeneous zirconia-alumina ceramics (due to lower specific gravities of mullite and aluminum oxide compared to zirconium dioxide). However, the obtained material did not have a distinct layered architecture (the composition changed smoothly in the volume of the cast body), and it was difficult to reach the same thickness of the layers of each composition in every casting trial. Minor micro-cracking was observed in the interior of some plates when they were cut for structural examination, which indicates reduced process repeatability and structural consistency. Ballistic testing of the graded plates did not show real improvement over monolithic alumina ceramic plates.

In the case of necessity to reach more discrete transitions of compositions within the body with a distinct layer-by-layer architecture, some other more complicated processing techniques for FGMs were discussed elsewhere [22,35,36,37,38,39,40]. However, not all the processing options may be suited for armor, especially when the armor components with complex shapes and large sizes are required.

For the FGM, the technologies based on consequent lamination of rather thin tapes (from below 1 mm to a few mm thick) can be considered. The tapes can be prepared mostly by rolling (high shear compaction HSC) or by tape casting (“doctor blade”) techniques, with their assembly. Electrophoretic deposition (EPD) may also be considered for the laminated structures; however, this process allows the formation of the structure with significantly thinner layers. In addition, EPD is not well suited for the manufacturing of larger-sized components with remarkable thickness, like armor products, especially for high productivity. The prepared individual tapes (made either by rolling or tape casting) are cut for required dimensions and shapes using either conventional knives or blades or, for higher quality and accuracy, laser beam cutting techniques. Furthermore, the cut tapes are stacked to assemble 3D bodies, which are then fired. However, the stacked laminates did not have good integrity after pressureless sintering because of organic binder decomposition, cracking, and fired material’s delamination, even after additional pressing of the stacked preforms before firing [4]. To avoid cracking and delamination of the final bodies at sintering, debinding of the green body and final hot pressing are very necessary.

The formation of FG ceramic composites with laminated structures made of different ceramic preforms but fired as a “single” body was explored in various studies using different tape preparation techniques [41,42,43,44,45,46]. Despite the recorded increase in toughness of the formed composites, notable issues related to structural instability and possible disbonding of the layers were observed. One of the major problems is related to high thermal residual stresses between the layers, which may lead to failure by delamination and edge cracking [20]. Thus, according to Menon and I-Wei Chen [44], composites based on the alumina and zirconia (Ce-TZP) tapes prepared by pressure-filtration, which were then rolled independently to thicknesses of 1–2.5 mm, were placed on top of each other and then rolled again, achieving a thickness reduction of ~50%. This operation was repeated several times, thereby reducing the total thickness and compacting the laminates. The green laminated structures containing multiple alumina-zirconia layers were fired at 1600 °C in an air atmosphere, achieving high-level sintered composites. However, due to residual stresses, the interface between two layers was unstable under large strain deformation, and, therefore, the laminated structure breaks down to a cellular structure. The instability primarily depends on the disparity of the yield stress of the layers, while the internal cracks, mostly in the zirconia layers, occurred due to differential sintering stresses and thermal expansion coefficient mismatch [44,45,46]. The laminates’ instability was greater as the layers’ thickness increased. Baskin et al. [46] recorded the tunnel cracks in the interfaces of laminates in the ceramics made by gel casting. The combination of equiaxed and textured alumina layers in the laminated composites allowed the internal stress reduction and deflection, and arrest of the cracking with stronger interfaces [47,48]. However, these authors used rather thin tapes (below 0.5 mm) for the laminates.

Considering non-oxide ceramic components, the preforms made of laminated tapes (layers), which may be prepared either by rolling (high shear compaction, HSC) or by tape casting, can be consolidated by pressure-assisting technology, e.g., by HP or SPS. Also, a porous 3D ceramic preform of a required shape can be infiltrated by molten materials followed by high-temperature thermal treatment that allows formation of the well-consolidated 3D composites through the reaction bonding mechanism. Metallic layers can also be utilized for the reaction bonding or joining mechanisms. Varying the layers’ composition and thickness, the FGM of desirable compositions and architectures can be obtained. Through this process, the layers with different porosities can be prepared to facilitate the preform infiltration and consolidation; this can be reached, for instance, by selection of starting powders’ particle sizes, binder selection, compaction at rolling, temperature at heat treatment, etc. Thus, the composites based on B_4_C, SiC, Si_3_N_4,_ and some others bonded by Si, Al, Mg, Ti, and some other metals can be obtained, and they can be well-suited for armor applications. Thus, the RBSC and RBBC laminated structures, where SiC and B_4_C layers were bonded by forming “secondary” SiC and residual Si, with flat or curved configurations (even simulated curved body armor plates), were demonstrated by Klosterman et al. [49,50], Windsheimer et al. [51], Caccia and Narciso [52], Wahl et al. [53], and some others.

## 3. Additive Manufacturing Possibility for Ceramic Armor

### 3.1. General Principles and Processing Options of Additive Manufacturing, Its Classification

**Figure 3:**
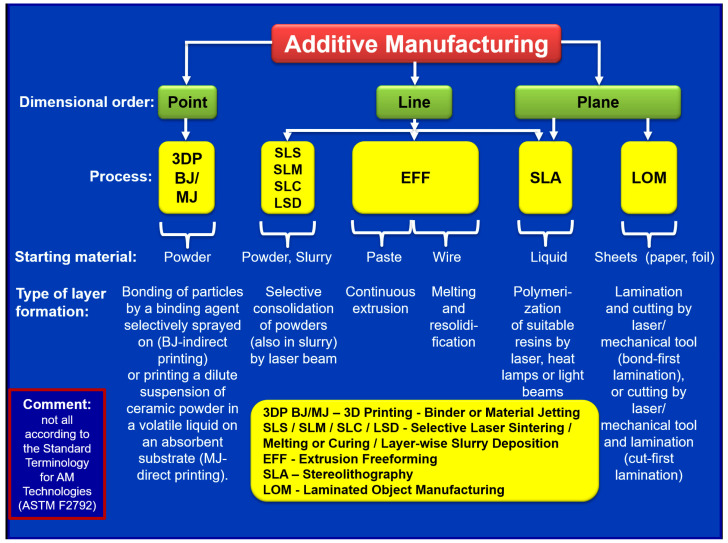
Classification of AM Processes According to Dimensional Order, Commercial Availability, Starting Materials’ State of Aggregation, and Type of Layer Formation.

Additive manufacturing (AM), i.e., “layer-by-layer”, has been paid more serious attention during recent years for the processing of different complex shape components utilizing computer-aided design (CAD). Mainly, the AM technologies allow a unique, relatively lower-cost opportunity to create components with new designs, especially with complex shapes, for their testing, evaluation, and further optimization before mass production when they are used correctly. They refer manufacturing of final components or rapid tooling and patterns required for the fabrication of final parts and components [54]. Figure 3 schematically shows the classification of AM processes for the manufacturing of ceramic materials according to the order of dimensions, availability, the aggregate state of the starting material, and the type of layer formation. According to ASTM F2792-12a (similar to ISO/ASTM 52900:2021) [55,56] and review [57], they include the following:

- Binder jetting (BJ), where a liquid bonding agent is selectively deposited to join powder materials;
- Material jetting (MJ), where droplets of the constructing material are selectively deposited;
- Directed energy deposition, where the focused thermal energy, like laser, electron beam, or plasma arc, is used to fuse the depositing materials by melting (SLS, SLM, SLC) or powder bed fusion, where thermal energy selectively fuses regions of a powder bed (LSD);
- EFF—material extrusion, where a depositing material is selectively dispensed through a nozzle or orifice;
- Stereolithography (SLA) or vat photopolymerization, where liquid photopolymer in a vat is selectively cured by light-activated polymerization;
- Laminated object manufacturing (LOM) or sheet lamination, where sheets of material are bonded to form an object. In fact, LOM should be considered as the additive/subtractive method due to the necessity to remove excessive materials (so-called “de-cubing”).

The number of different processing routes can be related to and depend on different factors, such as dimensions of the built component, equipment processing speed and productivity, dimensional accuracy and surface quality of the product, built component usage, material properties, necessity of post-processing, and cost. Several review articles related to the AM processes have been published in the last two decades (Table 1), specifically, the number of articles raised in recent years. However, the majority of the described processes may have only a limited application for industrial manufacturing of ceramic armor.

### 3.2. Challenges in Additive Manufacturing

Each AM process brings its own unique challenges; therefore, each process should be selected correctly depending on the required properties, dimensional, and application features. However, due to the layer-by-layer construction of parts, dimensional accuracy and surface quality remain critical in AM. For example, it is difficult to achieve precise layer deposition by distributing thin layers of powder in 3D printing techniques or to ensure a uniform application of a new layer of viscous ceramic slurry in stereolithography (SLA). A rough surface of such parts is a result of the “staircase effect”, and it is created by cutting the contour surface. Improved textures can be achieved by reducing a layer thickness or by secondary (post-firing) operations, such as grinding or polishing. However, these operations result in longer production times and higher costs. This is especially challenging for complex-shaped components, i.e., special grinding/polishing tools may need to be designed and fabricated. SLA offers the best surface finish compared to all currently available technologies, but it requires very expensive and limited raw materials (such as photosensitive resins). In some cases, to improve the surface quality and geometrical accuracy, a certain intermediate process step can be utilized. For example, the samples made of B_4_C/SiC/Si by binder jetting 3D printing were machined after the pyrolyzing step [53]. In this case, machining of pyrolyzed samples was much faster and easier than that of siliconized consolidated parts. Special care is required for cleaning the parts due to the necessity of using environmentally harmful solvents. In contrast, three-dimensional printing (3D printing) has limited surface finishing of the parts, but it allows a wide range of starting materials (in principle, any material available in powder form). Material recycling should also be considered in each specific case.

**Figure 4:**
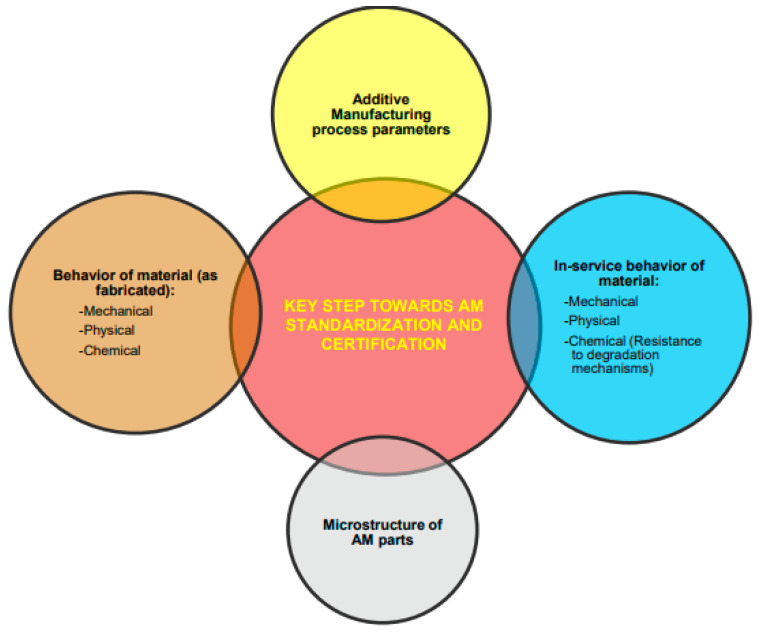

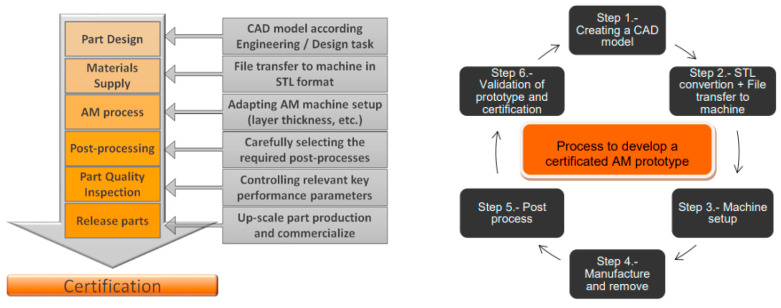
Certification Procedure for AM Parts, acquired from [71,81].

The resulting AM parts should meet certain desired property’s criteria, depending on specific applications. Emphasis should also be focused on new materials’ development in combination with suitable processing methods and optimal part designs. To ensure industry standards for AM processes, such as quality standards for producing parts or for sustainable and safe production, a six-step certification process was proposed (Figure 4) according to Á. Rodríguez-Prieto et al. [81].

### 3.3. Challenges and Potential Features of Additive Manufacturing for Ceramic Armor Components

Specifically for ceramic armor, the materials and components certification should include not only material’s physical properties determination and design optimization but also ballistic performance assessment of the armor system. Ballistic performance should be evaluated through actual ballistic testing using actual projectiles. This is important since all physical properties and performance strongly depend on ceramics’ microstructure and crack propagation features upon high-velocity ballistic impacts, which significantly differ from static mechanical loading. Processing influences on the design and structure of the ceramic components and possible defects, which may occur in production, and which will affect cracking and disintegration of the components under ballistic impacts. It should also be kept in mind that armor ceramic components need to be bonded with a backing material, and the bonding technology is related to design and required performance of armor systems.

**Figure 5:**
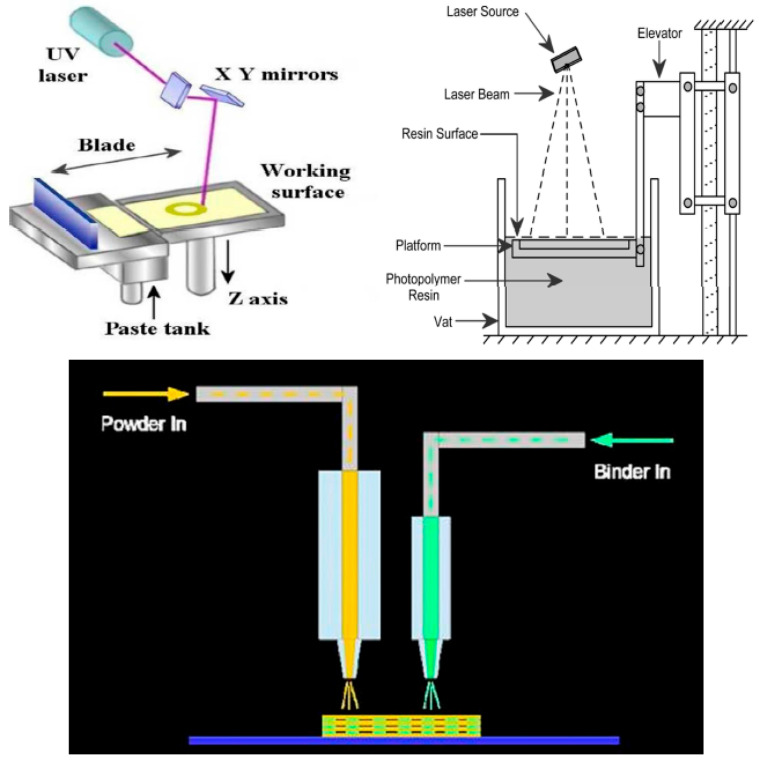
Schematic of the SLA Process Applied for Alumina Armor Discs [82] (**upper image**) and Schematic of the DIW (Robocasting) Process Applied for Alumina Armor Tiles [83] (**bottom image**).

During recent years, some works utilizing AM were conducted to obtain ceramic armor with “constant” structure and properties through the whole “body” with certain success, but these studies did not confirm the real benefit of AM over “conventional” forming processes [82,83,84]. Thus, Carton and Weerheijm [82] compared alumina ceramics commercially produced and 3D-printed (stereolithography was used as the processing route) (Figure 5) and fired at 1650 °C. Although both materials had similar mechanical properties (while the density of 3D-printed ceramics was lower), the authors recognized that ballistic performance (tested using a 7.62 mm projectile with a steel core; velocity ~800 m/s) of the 3D-printed ceramics was lower (~2 times lower dwell time and ballistic efficiency) compared to the commercially produced alumina tiles. Larger variations in the obtained data for the 3D-printed ceramics were also observed. In another study, Jones et al. [83] ballistically tested alumina ceramic tiles (90 × 90 × 8 mm), which were also produced by different routes, using a 12.7 mm AP M2 (with a steel core) projectile (velocity ~850 m/s). They found that the ceramics made by direct ink writing DIW (robocasting) (schematic in Figure 5) performed better than the ceramics obtained by pressurized spray deposition; however, the performance of the tiles made by the mentioned AM technologies became reduced compared to the tiles made via “conventional” isostatic pressing. Thus, a significantly higher depth of penetration and greater disintegration and fracturing were recorded for the 3D-printed tiles. Ballistic performance of alumina ceramics obtained by the SLA process also was not higher than conventionally produced alumina ceramics when the ceramic samples were tested using 7.62 × 39 mm steel-core threats (velocity ~850 m/s) [84]. The comparison of alumina ceramics made via SLA and conventionally produced ceramics containing 95-wt.-% Al_2_O_3_ showed that the former had higher porosity, less consistent structure, and lower physical properties.

We believe that the inferior performance of the AM-produced ceramics may be related to excessive flaws and micro- or even macro-defects within the “single” layer’s structure or at the layers’ interface. These defects may be a sequence of, for example, (a) not very successful dispersion of the ceramic slurries, or (b) poor compaction of the layers containing excessive amounts of entrapped air and/or a necessity to use enlarged contents of organic binders in the suspension or paste resulting in excessive gas emission at firing, or (c) a combination of the listed points at the conducted experimental works. Poor compaction and/or excessive gas emission at firing lead to worse ceramic materials’ consolidation, specifically at the layers’ interfaces (even with possible delamination). Thus, buckling and related deformation within the “single” layer may be one of the reasons for the occurring flaws and lack of densification. For the ceramics made via LOM, the sides and edges have lower densification and side cracks, and these issues also reduce the components’ production yield, performance, and applicability. Thus, according to our studies, the planar oxide ceramics obtained by the rolling (HCS) technology had side cracks, which propagated from the sides to the middle. This process included: (i) formation of thin, rather flexible, sheets; (ii) stacking of these sheets; (iii) rolling of thick laminates using significantly larger rolls, which provided a significantly higher compaction under higher pressure, with cutting the uneven edges; and (iiii) final pressureless sintering of the compacts. These laminated ceramics also had uneven densification with lower density in the side areas. While delamination can be minimized using larger diameter rolls providing a higher compaction, the side cracks are enlarged at higher compaction forces. In this case, excessive de-cubing is required. These uneven compaction and side cracking are related to inconsistent pressure distribution from the middle to the perimeter at rolling (the soft material “flows” under pressure from the middle to the sides); uneven compaction results in uneven shrinkage at firing of these compacts, their distortion, and firing-induced cracking.

To minimize these issues, an additional processing step enhancing green body compaction, which should also reduce flaws and internal defects, can be utilized. In this case, pressure-assisted firing, such as HP, can be employed, but this route may increase the processing cost. Thus, Wahl et al. [53] increased density and reduced internal micro-defects in the 3D-printed compacts prepared from B_4_C and carbonaceous powders utilizing isostatic pressing. Furthermore, the obtained compacts were infiltrated using liquid silicon with the formation of reaction-bonded boron carbide ceramics. The obtained RBBC ceramics had practically zero open porosity and a high level of mechanical properties (e.g., hardness ~20 GPa, flexural strength ~355 MPa, Young’s Modulus ~340 GPa). This approach may be successfully employed for AM-processed ceramic armor.

Comparing “conventional”, well-established technologies, such as slip casting, pressing, and some others (e.g., based on the reaction bonding processes) with AM technologies, the former provides not only adequate properties and performance but also productivity, which is significantly higher than AM can provide at present. In fact, the major benefit of AM is the possibility of obtaining specific “complex” product configurations, which are challenging for most “conventional” technologies. In contrast, the major armor components, such as body armor plates and tiles for vehicular, aircraft, and structural ballistic applications, have rather simple shapes, and these plates and tiles are required and commercially produced in high volumes. However, for specific situations with special ballistic requirements focusing on the armor systems of lower weights and for unconventional components’ designs, certain AM technologies can be considered and employed. The involvement of different ceramic materials with their own properties and structures, creating the graded architecture (with tailored layers’ thicknesses and/or with particular locations of the “zones” with required structures and properties), will allow a unique opportunity to create lower-weight armor components. Increased ballistic performance may also be attained by modifying the localization of the energy of the shock waves and the ceramic fracturing mechanism through the graded architecture.

Although AM is widely considered for numerous ceramic components for various applications, including for armor, it is necessary to analyze the AM positive and negative features, especially from more “practical” viewpoints. This paper reviews, for the first time, the AM processing routes for ceramic armor components not only from the potential “attractiveness” of this technology, but it also analyzes these manufacturing possibilities, indicating the technology routes and structural features, which may have higher potential for ceramic armor. The use of certain AM routes specifically for functionally graded ceramic armor (FGCA) is proposed, considered, and emphasized, for the first time, as more promising.

## 4. Possibility of Additive Manufacturing for Functionally Graded Ceramic Armor

### 4.1. General Possibility of Additive Manufacturing for Functionally Graded Ceramic Armor

**Figure 6:**
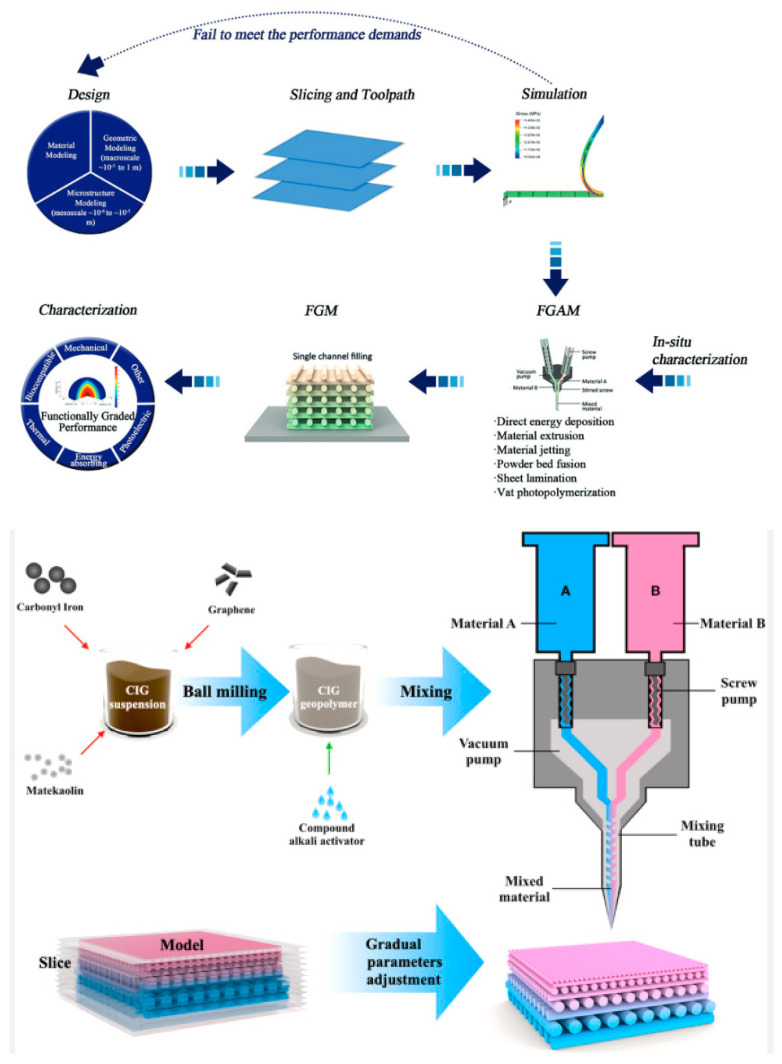
Principal Schematics of Processing and Characterization of FGM via AM (FGAM), acquired from [88,90].

Additive manufacturing (AM), as a versatile technology where different materials and shape options can be involved, may be considered as one of the most reasonable routes for functionally graded ceramic materials. Several review articles describe different AM options for dense and porous materials, from metals to polymers and ceramics, for a variety of FGM applications [85,86,87,88,89]. Although AM includes different processing paths, only some of them can be considered as feasible for functionally graded ceramic armor (FGCA) component production. As an example, a principal schematic of FGCA using the AM processing options is presented in Figure 6 [88,90].

The important condition of ceramic-based FGM with high integrity is the minimization of cracking and delamination occurring at the layers’ interface. It is known that internal stresses, particularly in multi-layer bodies, can occur at sintering due to external constraints or differential densification. These stresses can lead to significant changes in the strain rate, distortion, and damage of the sintered parts. In order to minimize these issues, the involved materials have to have the same drying rate and firing temperature and similar sintering rates, as well as the obtained layers need to have similar shrinkages at the firing. In some cases, hot pressing of the prepared FGM green body can be employed; however, pressure-assisted sintering may not be very desirable due to cost and productivity points of view, especially for large-sized components. Significant attention needs to be addressed to theoretical and experimental studies on constrained sintering and stress conditions.

**Figure 7:**
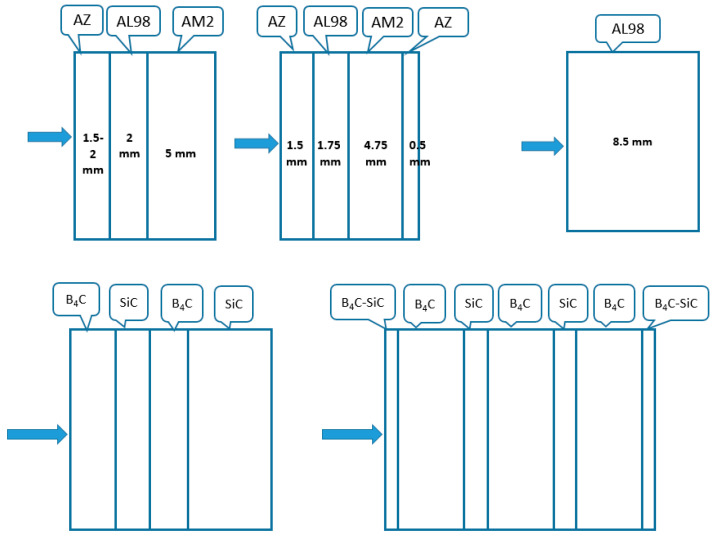
Functionally Graded Alumina-Based Armor Ceramics (**upper**) and B_4_C–SiC Armor Ceramics (**lower**)—Principle Design Examples (arrow indicates the projectile direction).

One of the design options of FGCA based on Al_2_O_3_ may consist, for example, of high alumina (AL99.7) or alumina-zirconia (AZ) ceramics at the front (strike) face as these materials provide the highest hardness (~1560 kgf/mm^2^ or ~15.6 GPa HV10) and remarkable fracture toughness (~4 MPa.m^0.5^ for AZ) compared to other alumina ceramics. The properties of the mentioned ceramics are shown in Table 2 [3,13]. The principal architecture (schematic) of this option is proposed in Figure 7. The ceramic materials for this design (AZ, alumina, and alumina-mullite ceramics) can be prepared using the same types of slurries with similar pH levels at colloidal processing. The hardest ceramic layer at the front should intensify the projectile breakage and disintegration at ballistic impacts. Behind the front ceramic layer, alumina ceramics with the compositions of AL98.5 and/or AL98 and alumina-mullite ceramics AM2, which are lighter and have lower hardness than AZ, can be arranged. The thickness of the front hard but heavier layer may be rather small, like 1.5–2.5 mm, while the thickness of AM2 ceramics with significantly lower density due to high contents of the mullite phase can be as 4.5–6 mm. Because of the graded architecture, the total weight of the ceramic body could be reduced compared to non-graded alumina ceramics. In this design, even the use of the AZ layer (~0.5–1 mm thick) with elevated toughness and good ballistic energy dissipation (i.e., with a rather low value of the criterion D) in the back of the ceramic body can provide a weight reduction compared to alumina ceramics. High multi-hit ballistic performance of these composites is also expected.

However, the total thickness of the front ceramic layer involving different ceramic layers needs to be considered in the armor system design and manufacturing. As was shown earlier [4], if the thickness of the front ceramic plate is sufficient (e.g., ~7 mm or greater for body armor plates), the cracking related to shock wave reflection was rather minimal, and the erosion of the projectile was substantial, which provided adequate ballistic performance. However, if this thickness is small (below 4–4.5 mm), the cracks related to shock wave reflection were intensive, the projectile erosion was insignificant, and, as a result, the ballistic performance was not high enough.

Considering this compositional and processing approach (e.g., jet printing or direct ink writing), the same temporary organic binder with the same content in a slurry or in a paste can be used for processing of different ceramic layers, and no polymeric interface in the green state needs to be formed. This will minimize the difference in shrinkage of the layers, thereby also minimizing the related micro-cracks and delamination issues. Since the mentioned ceramic materials can be fired at the same temperature using the same firing profile (pressureless sintering) and since they have rather close values of shrinkage factors, possible internal thermal stresses and delamination that may occur during the firing would be insignificant. Moreover, due to a slightly higher shrinkage factor of AZ (compared to alumina AL98 and AM2 ceramics), especially when the AZ layers are on the front and the back surfaces, the ceramic body will be under compression. This will be more beneficial for the armor ceramics’ behavior at ballistic impacts. However, to minimize the possible occurrence of micro-cracks and residual stresses at the materials’ interfaces, the shrinkage of the involved materials needs to be tuned. This can be attained, for instance, by the directional tuning of ceramics’ and suspensions’ compositions.

It should be noted that the particles’ compaction within and between the layers formed by the majority of AM methods may not be as high as commonly achieved by slip casting or pressing. Also, excessive porosity and flaws or a laminar structure within ceramics, and even possible local delamination, may occur. These structural issues may be rather critical, specifically when actual armor components are produced. Comparing the AM-based deposition of the layers through the nozzle (like in DIW or robocasting) with the “conventional” extrusion process, the latter process provides significantly higher particle compaction due to the applied higher pressures in the extruder and due to the extrusion die design. To compensate for possible lower consolidation of the AM-processed FGCA, an additional compaction of the green body could be applied. This can be realized by uniaxial pressing of the FG green compacts in the case of simple planar armor components or by cold isostatic pressing (e.g., for curved body armor plates and for more complex shape components); the latter option would be even more effective in providing a higher degree of compaction. This additional “post-compaction” will also minimize possible internal defects between the layers made of different materials with different compaction levels and with different shrinkages at the “green” (unfired) state. However, this additional step will lead to the processing cost increasing. The ceramics’ firing temperature increase and/or the increase in the soak time at firing can be another possible route, but this route, which increases the process cost, may not be a complete solution. Because of this, physical properties of the AM-processed ceramics may not be as high as achieved by the “conventional” technologies (the properties of some slip cast and pressed ceramics formed through pressureless sintering at a temperature below 1550 °C are presented in Table 1). Another option to compensate for the occurring structural defects, which potentially reduced armor ceramics’ performance, can be either a slight increase in the ceramic body thickness or an increase in the backing material thickness (for example, an increase in the number of Kevlar plies) in the armor system. However, this option would lead to the thickness and/or weight increase in armor with its cost increase, which would not be highly desirable.

A similar approach can be used for the design and processing of non-oxide-based ceramics, e.g., with B_4_C–SiC compositions (Figure 7); in this case, pressure-assisted sintering (e.g., HP or SPS) can be employed, although pressureless sintering can also be possible. Specifically, the high brittleness of B_4_C ceramics, which negatively affects its ballistic performance under multiple hits, may be significantly reduced by toughening and tailored distribution of residual stress through structure lamination. As an example of potential FGCA, Pelz et al. [91] prepared small discs (~25 mm dia.) based on B_4_C and SiC layers using a direct ink writing technique, which were consolidated by hot pressing.

### 4.2. Consideration of Robocasting or Direct Ink Writing for Functionally Graded Ceramic Armor

**Figure 8:**
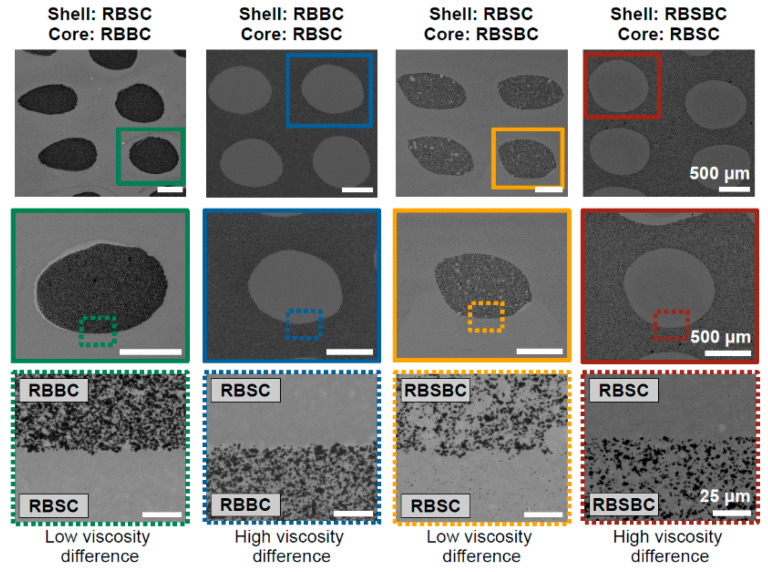
Microstructures of the Core–Shell RBSC, RBBC, and RBSBC Composites [96].

**Figure 9:**
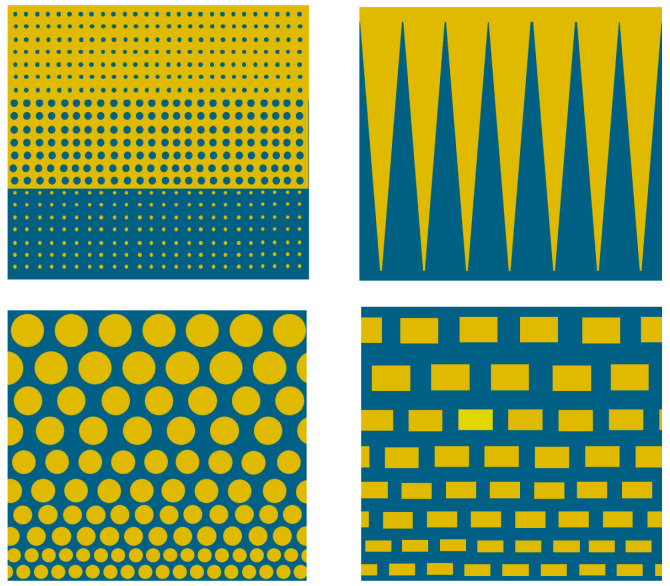
Schematic of Possible FGM Designs Obtained by Robocasting, e.g., Co-Extrusion, Using Different Nozzle Shapes.

Robocasting or direct ink writing (DIW) can be considered as another potential AM technology suited for FGM components, including for ceramic armor. In many cases of robocasting of advanced ceramic materials, a ceramic paste is extruded through a small-diameter nozzle, controlled by a specially developed computerized program, to obtain the desired shapes [92,93,94,95,96]. In fact, in robocasting, which may include extrusion and co-extrusion, the nozzles of different sizes and shapes (e.g., round, oval, square, rectangular, etc.) can be used [95]. Thus, Gradaus et al. [94] demonstrated the core–shell structures based on RBSC, RBBC, and reaction-bonded silicon carbide-boron carbide (RBSBC) composites. In this work, the pastes with high solid contents also containing necessary carbon were used for co-extrusion, and post-liquid silicon infiltration provided resultant ceramics’ formation and consolidation. The microstructures of the composites fabricated by this route are shown in Figure 8. Figure 9 schematically shows an example of possible FGM designs using nozzles with different shapes. Therefore, robocasting, particularly co-extrusion, can be very interesting for the fabrication of FGMs of different compositions, where shells’ and cores’ compositions in the ceramics may differentiate.

It is important to mention, once again, the possible generation of residual stresses caused, for example, by the thermal expansion mismatch of the phases in the fabricated materials. Residual stresses can significantly affect the mechanical properties of the resulting composites [97], and the design of the ceramic structures with a certain stress distribution may be important for the ceramic armor with improved ballistic performance. Again, it should be kept in mind that the compaction in the extruded pastes in robocasting is reduced compared to the “conventional” extrusion, as mentioned above. Therefore, an additional post-compaction step can be applied to improve ceramic components’ densification and to reduce internal stresses within the components.

In the case of armor ceramics, which can be processed through the reaction bonded mechanism, like RBBC and RBSC, the FG porous preforms can be processed AM, and then these preforms can be siliconized with consequent pressureless sintering. For armor SiC–Al_2_O_3_ and SiC–Si_3_N_4_–Al_2_O_3_ ceramics, which can be consolidated through the reaction-bonding mechanism but without infiltration and without specially controlled firing atmosphere [3,13], the required specially selected particle size distributions may also be realized by certain AM paths, e.g., by direct ink writing or jet printing. The creation of the gradient grain size distribution in these ceramics will be helpful for reducing crack propagation, as well as for facilitating the erosion of the projectile. Thus, the layers with the coarser grains may be arranged in the middle of the ceramic body or at the back. Since these ceramics do not shrink at firing, the graded structure (obtained through the modified grain sizes) would not have possible stressing problems commonly associated with different firing shrinkages of different ceramic materials.

**Figure 10:**
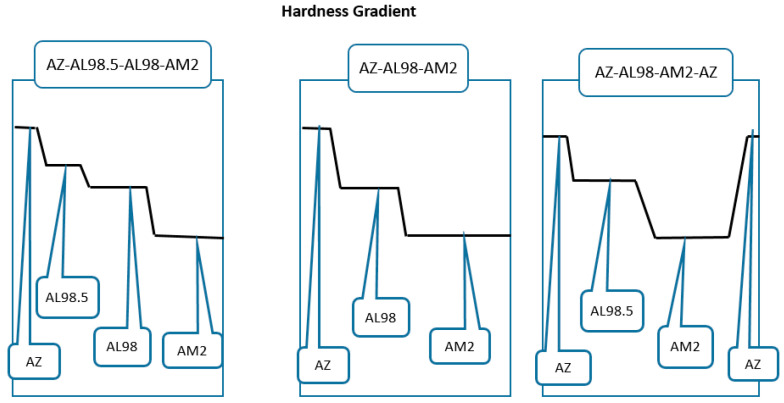
Hardness Level of Functionally Graded Alumina-Based Armor Ceramics—Schematic.

The concept of FGCA made using AM will allow for increasing failure strength in ceramics by creating a surface layer with compressive stresses, which will reduce the surface cracks and failure stresses. This can be considered beneficial for the product integrity according to the studies [98]. The residual stresses between the “neighbouring” layers may be estimated through the values of Young’s moduli and Poisson’s ratios of each material involved in the design, as well as taking into consideration the thickness of the layers [25]. Since the forming residual stresses occur at sintering, coefficients of thermal expansion and temperature gradients between the sintering temperature and the temperature, when the integration between the layers occurs, need to be involved in the residual stress estimation. Similarly, it is possible to arrange the layers with a certain transition of hardness and toughness from the front to the back surface. As an example, a schematic of possible hardness gradients in this type of ceramic armor design can be seen in Figure 10.

### 4.3. Consideration of Laminated Object Manufacturing for Functionally Graded Ceramic Armor

**Figure 11:**
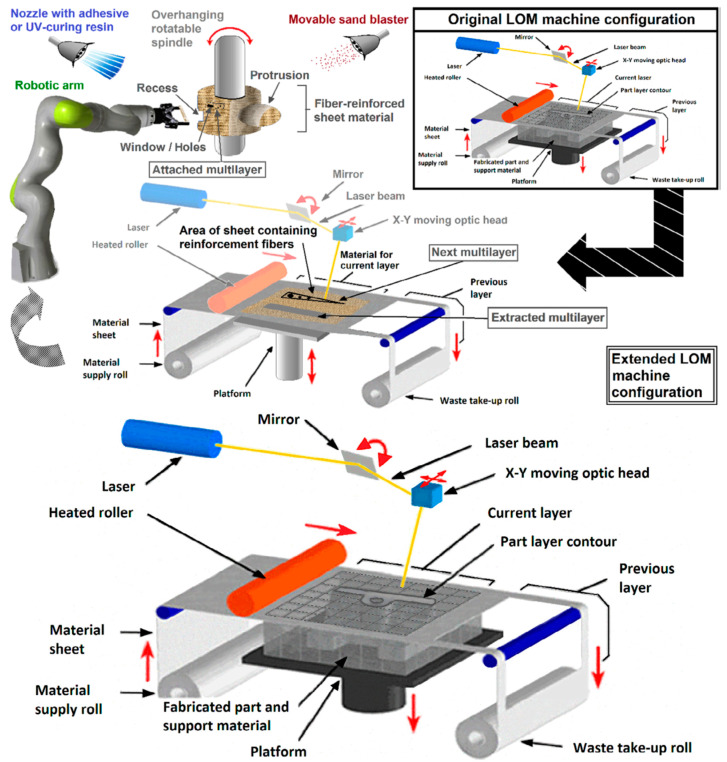
Principle Schematic of the LOM Process through Rolling, adopted from [71] (red arrows show the continuous flow of sheet material).

**Figure 12:**
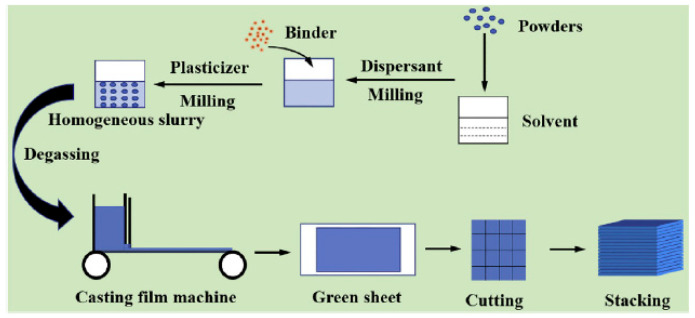
Principle Schematic of the LOM Process through Tape Casting (adopted from [69]).

**Figure 13:**
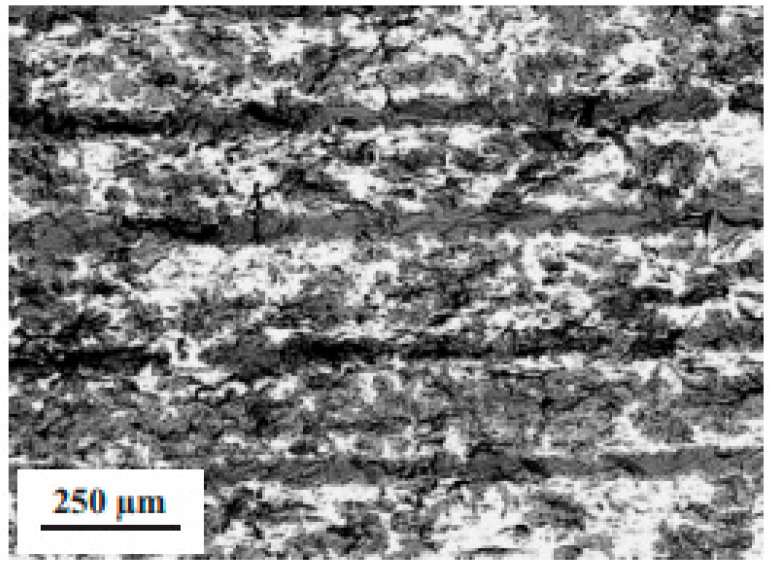
Microstructure of Laminated Si–SiC Composites (adopted from [99]).

Considering non-oxide ceramic armor components, the laminated object manufacturing (LOM) path, as the AM (layer-by-layer) option, should also be outlined. LOM is a highly promising, versatile method for the fabrication of parts with various dimensions ranging from millimeters to meters [71,99]. This process is schematically shown in Figure 11. The LOM process was initially developed for the construction of paper-based multi-layers or multi-layers made of thermoplastic polymer films. Two different methods have been developed for LOM: the bond-first (named as “bond-then-cut”) lamination and the cut-first (named as “cut-then-bond”) lamination. According to Liao et al. [100], the amount of waste material, which has to be removed after LOM with the cut-then-bond method, was 30–80% less compared to the bond-then-cut method (de-cubing). In both cases, a laser beam or a mechanical knife can be successfully employed as a cutting tool. In this path, as mentioned above, the sheets or layers (e.g., tapes, foils, etc.) can be prepared by rolling (high shear compaction, HSC). Tape casting may also be utilized, but with less manufacturing capacity, since it may be suited for rather thin tape preparation (Figure 12 [69]). The prepared layers should be stacked and cut to make the required components’ configuration and thickness, and then the obtained preforms should be consolidated, reaching a final body. As mentioned above, the FGCA component consolidation can be achieved by a pressure-assisted route (HP or SPS), significantly enhancing densification with minimal delamination and internal cracking issues. Infiltration (non-reactive or reactive) with a molten material, e.g., Si, which may also provide the reaction bonding (joining) mechanism, as demonstrated by many researchers [6,14,15,51,71,99,100,101,102,103,104], can be successfully employed to prevent possible delamination. The example of the obtained microstructure of the laminated Si-bonded SiC ceramics is shown in Figure 13 [99].

**Figure 14:**
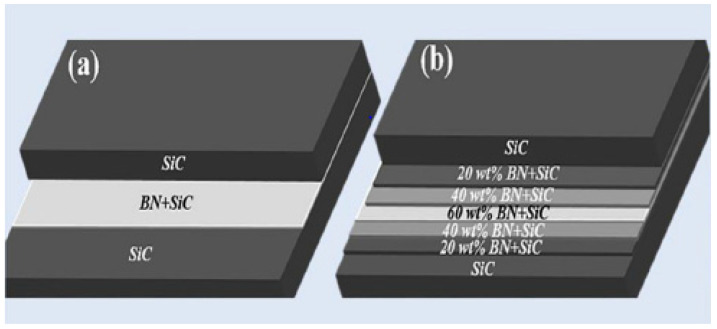
FGM Based on Monolayers (**a**) and Gradient Layers (**b**) [105].

This technology allows producing the FGM based on monolayers and gradient layers. Thus, Sun et al. [105] created the components made of SiC/BN layers prepared by tape casting, where, as a special option, the intermediate BN/SiC layer consisted of a number of thinner layers with variable BN contents (from 20 to 60 wt.-%) (see Figure 14). The sinterability of the SiC layer was attained by the Al_2_O_3_–Y_2_O_3_ doping. Although high-temperature pressureless sintering (1900 °C in Ar) provided a high consolidation level, reducing potential processing cost (compared to pressure-assisted sintering), the cost remains rather high. The obtained ceramics demonstrated high mechanical properties, e.g., bending strength as ~300 MPa and especially fracture toughness as ~8.5 MPa.m^1/2^. As expected, the crack propagation in these multi-layered composites was delayed, providing the energy absorption. Specifically, fracturing in these components occurred through delamination cracking, crack kinking, and crack deflection; the delamination cracking occurred inside the BN layers or at the SiC-BN interface.

**Figure 15:**
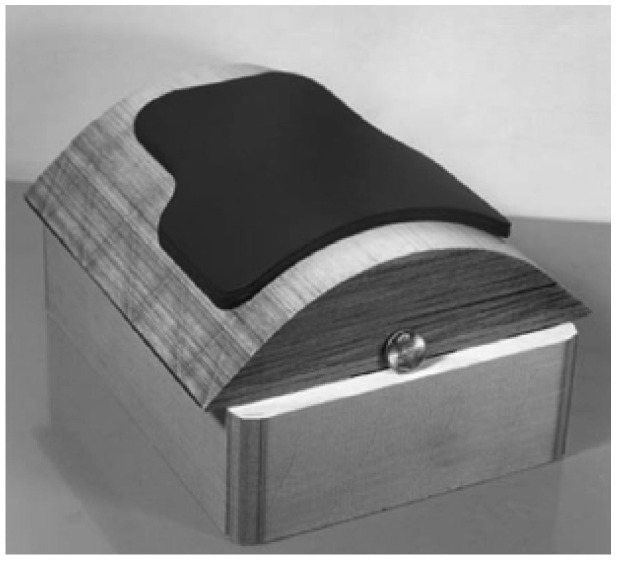
Curved Layer of the Monolithic Body Armor Immediately after LOM Processing [106].

In order to fabricate curved components, which are common for body armor, using LOM, the SiC-filled tapes were prepared by a standard doctor blade tape casting process, and then they were stuck to a paper mandrel and formed to the desired curvature (Figure 15) [106]. The tape casting slurry consisted of a mixture of bi-modal SiC with large-size (60 μm) and small-size (2–3 µm) powders, carbon black, and graphite powders dispersed in a polymer binder. The binder content in the tapes was 15–20 wt.-%, and the prepared tape thickness was 250 μm. These authors showed that the curvature was matched to the part design, facilitating subsequent waste removal by minimizing the areas of overlap. The LOM-produced green bodies were first pyrolyzed at 325 °C under pressure to prevent delamination. Next, the pyrolysis was completed at 700 °C in an argon atmosphere. Upon a silicon infiltration step at 1600 °C, the green bodies were converted into dense SiC parts. The obtained monolithic SiC ceramics had a bending strength of 142–165 MPa measured according to the 4-point bending test.

As mentioned above, AM can also be applied for the fabrication of tools or supporting structures, such as paper (or tape) mandrels. For these purposes, different AM methods, for instance, LOM, 3DP (Binder Jetting), etc., can be used.

### 4.4. Analysis of Possible Structural Features of Functionally Graded Ceramic Armor Processed Through Additive Manufacturing

The ceramics with designed laminated structures made of different materials may be a more interesting approach to achieve even a greater internal stress reduction at the layers’ interfaces, as well as for a manageable weight reduction. For instance, a few layers (0.5–2 mm thick) of B_4_C and B_4_C–SiC ceramics prepared through high shear compaction (rolling) with consequent hot pressing have the potential to minimize the interface issues [24,25]. This work was focused on the modification of crack propagation due to the laminated graded structure to improve the composites’ ballistic performance. However, the weaker point of this design and manufacturing is the layers’ interface due to high contents of temporary organic binder involved in the processing; in addition, this process is less productive. Similar results were obtained by Hong et al. [107], where the SiC-based tapes were prepared by tape casting (“doctor blade”), and the laminated preforms went through the debinding step first and then through hot pressing. In this work, the “matrix” and “interface” tapes had compositions (wt.-%) of 92 SiC—8 Al_2_O_3_/Y_2_O_3_, and 46 SiC—46 h-BN—8 Al_2_O_3_/Y_2_O_3_, respectively. The interface layer(s) (SiC–BN) in the laminated ceramics, as demonstrated above (Figure 14 [105]), promoted the material’s energy absorption ability. These FGCA demonstrated reasonable ballistic performance with the reduced back face signature (BFS) depth compared to solid-state sintered SiC ceramics. The BFS was reduced by increasing the number of interface layers in the gradual-layered ceramic composites. The ballistic testing was conducted for flat 5.5 mm thick tiles bonded with UHMWPE backing in conjunction with an armor vest, using a 5.8 × 42 mm projectile with a steel core (velocity ~900 m/s).

With regard to the laminates prepared from different oxide/non-oxide compositions, the Al_2_O_3_–SiC composites, reported by [108], and the ZrO_2_–ZrCN/Si_3_N_4_ composites, reported by Li et al. [109], can also be mentioned. In the latter report, the tape cast and reactively hot-pressed composites demonstrated promising performance after the split Hopkinson bar impact testing. Multi-reflection and attenuation of stress wave at interfaces were the main reasons for this good energy absorptivity upon impact. For the SiC/B_4_C/Si FG armored bodies, the tapes of SiC and B_4_C (in some cases, adding selected carbon contents) of different thicknesses can be stacked and shaped using LOM machines, pyrolyzed and finally infiltrated with molten Si. In this case, the possible formation of residual stresses can be taken into account. As mentioned above, in many cases, water-based organic binders are used in the AM processes, and high contents of these binders may be one of the causes of elevated porosity and flaws after the binder burnout, especially for oxide ceramics. However, for non-oxide ceramics and composites produced through Si infiltration, which is commonly conducted in reducing or neutral atmospheres, the residual C contents can be the source for the formation of secondary SiC due to reaction bonding. In this case, the use of non-water-based organic binders (with remarkable contents of C) may be reasonable if the involved ceramic suspensions have minimal entrapped air and “workable” viscosity, providing good flows.

**Figure 16:**
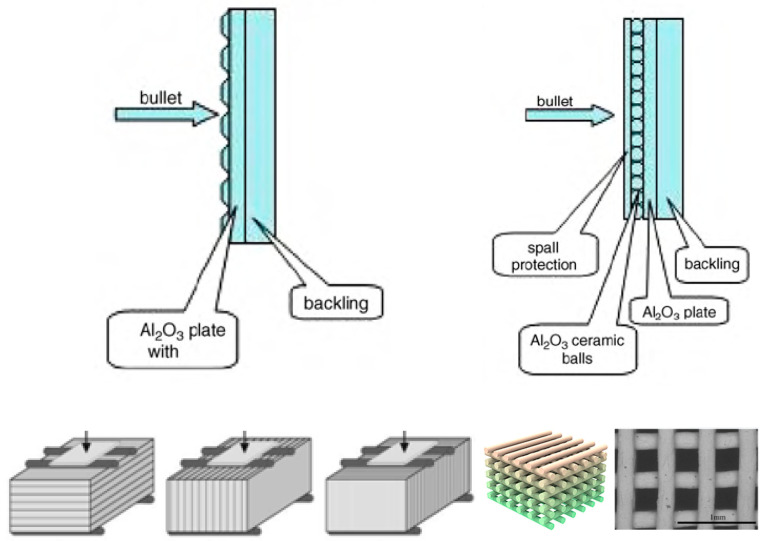
Reinforcement Options of Ceramic Armor, which May Be Attained by AM.

The AM technologies can be applicable to design and produce armor components with variable thickness in one body. In particular, the increased thickness of the edges for the armor plates’ or tiles’ perimeter can be made to improve their ballistic performance, since the cracking, upon the ballistic impact, is more pronounced at the edges. Moreover, the edges with an elevated thickness can be made using the harder material with a slightly higher shrinkage factor to create a confinement of the main body to reduce fragmentation. Similarly, the front (strike) surface with the multiple “nodes” with the conical or round shapes or the ball-like shapes, where the balls or spheres are connected (as shown in Figure 16), can be produced to modify the impact angle. This can lead to altering the direction of the crack propagation and ballistic energy dissipation at the ballistic impact and, finally, to increasing ballistic performance. Although this design is possible by more “conventional” processing routes (e.g., by casting, injection molding, etc.), the molds to obtain this configuration become more complicated. Moreover, the reinforcement of the armor ceramic body can be performed, employing AM with the appropriate computer programming. This may include the designs with a “mesh” (honeycomb) or “bars” with different orientations or “platelets” inserted into the middle of the main body; the reinforcing ingredients can be produced with ceramic compositions different from the matrix (Figure 16).

A thin ceramic or glassy layer or coating on the major armor ceramic body, specifically, with a higher shrinkage at firing, can create the compressive-stressed conditions, which may also be beneficial for ballistic performance by reducing crack propagation. While a thin ceramic coating layer may be produced using different thermal spray processes [110], a glassy or glaze layer may be produced using “conventional” spraying of the coating suspension or by dipping the component into this suspension, followed by low-temperature heat treatment, resulting in softening, liquefying, and even distribution of the consolidated compound. In any case, a proper adhesion of this coating layer to the “main” component must be provided. Alternatively, a thin metallic coating can be formed around the main ceramic component, providing confinement to reduce fragmentation upon multiple ballistic impacts.

Highly porous ceramic structures fabricated by AM and their infiltration with polymeric compounds or metals may also be interesting options for the armor system designs. The armor systems, which included reticulated ceramics infiltrated with polyurethane, demonstrated high ballistic performance [111], and the composite materials fabricated with honeycomb meshes or bars with different orientations using AM technologies and infiltrated with special polymeric compounds may be even more versatile armor systems with controllable designs. Liquid metal infiltration can also be applied, just as Halverson et al. [112] proposed for the preparation of B_4_C–Al composites. Employing the AM technology, the components with enlarged grain sizes and residual open porosity at the back surface of the consolidated bodies can be formed. This approach was successfully employed by Wang et al. [31] and Zhang et al. [113]. In the latter study, continuous and stepwise graded multi-layer B_4_C–Al composites were produced by melt infiltration of the preforms prepared from B_4_C powders with different particle sizes. This option creates the opportunity for the post-infiltration (reactive or non-reactive, pressureless or pressure-assisted) with metal (like aluminum or titanium alloys), providing additional ductility and toughness of monolithic ceramic–metal composites. Since infiltration of molten metal into pores and cells in ceramic surfaces and wetting of ceramic surfaces by molten metal are critical for proper adhesion and a strong ceramic–metal interface, the AM-designed structures with larger cells would be more favorable (compared to the structures with large pores).

All of these design variations can also enhance ballistic performance, especially in multi-hit situations, through modification of the stress conditions, shock wave, and the crack propagation. These types of armor can be considered for personnel, vehicular, aircraft, and structural ballistic applications. Specifically, they could be beneficial for modular applique armor panels for vehicular and aircraft systems, where simple flat tiles or plates can be replaced by specially designed lower-weight components and complex-shaped parts.

### 4.5. Potential Technological Routes to Obtain Functionally Graded Ceramic Armor by Additive Manufacturing and Possible Structural Features of Functionally Graded Laminated Components

Despite a unique possibility of AM technologies to shape components with very intricate configurations, including components with configurations that were not produced before, it needs to be kept in mind that these components should be fired without distortion. As mentioned above, manufacturing productivity and processing costs should also be kept in mind. Moreover, the armor system design should consider not only the ceramic body design and its manufacturing, but also the necessity to bond the ceramic body with backing materials and wrapping of these components with fiber-based prepreg, confinement, and spall protection layers, etc. In addition, the armor ceramic body should have surface finishing suitable for bonding with backing, and if this ceramic body has an intricate shape, a special surface machining procedure may be required, and the related grinding tools will need to be designed and fabricated. Because of these factors, the “attractive” biomimetic armor design, even with a potential use of AM technology, as considered by Islam et al. [114] and Li et al. [115], is still hardly viable for now and for the foreseeable future.

Considering the manufacturing of FGCA, colloidal processing is highly desirable to attain a proper homogeneity of ceramic mixtures and suspensions. Widely available and commercial sources of starting materials should be utilized. Starting materials and suspensions’ compositions should provide possibly minimal linear and volume shrinkages to reduce distortion and cracking associated with ceramic components’ firing and structure consolidation. Although this is important for any ceramic technologies, it is especially critical for AM processing routes and for FG ceramics. In this regard, the AM technologies based on preceramic polymers, like those proposed and discussed by Han et al. [116], would not be well-suited, since the “preceramic polymers” approach is based on very limited starting material options (Si-organic polymers). Furthermore, the obtained ceramics experience high firing shrinkage (from ~30 to ~60% that is, basically, significantly high compared to ceramics based on “conventional” starting materials and technologies), resulting in significant distortion and associated cracking. These negative features will be especially critical for FG ceramic components involving materials with different compositions.

**Figure 17:**
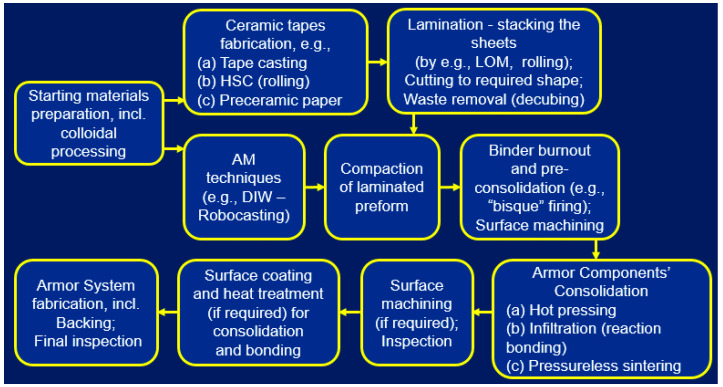
Simplified Schematic of Functionally Graded Ceramic Armor Using Two AM-Based Processing Approaches (LOM and DIW).

While, as mentioned above, “conventional”, highly productive forming processes are successfully employed for ballistic armor, certain AM technologies can be recommended specifically for FGCA with intricate shapes and dedicated applications where ballistic impact energy dissipation, crack propagation, and stress distribution could be managed. The proposed general schematic of FGCA manufacturing is presented in Figure 17.

At the FGCA design and manufacturing, the residual thermal stresses, which can commonly occur during the high-temperature consolidation step, specifically at the cooling stage, need to be considered. These residual stresses in the layered ceramics depend, as indicated by Orlovskaya et al. [25], on the elastic properties (Young’s moduli, Poisson’s ratio) of the ceramic layers of different compositions, their coefficients of thermal expansion, temperature gradient, as well as on the thickness of the layers and total thickness of the ceramic body. It should be kept in mind that these stresses may be localized, specifically at the periphery or at the edges of the produced components, and the stresses within the components may vary, depending on the components’ shape and dimensions, from tensile to compressive. The FGCA design should be focused on the localization of the energy of the shock waves occurring under ballistic impact. This energy localization, mostly within the ceramic body, can create the “reverse Hertz cone” upon impact. As was shown earlier [4,117], the layer consisting of a polymeric compound filled with lightweight solid or porous ceramic grits, which separated a front armor ceramic plate from a backing material, promoted the absorption of kinetic energy of the projectile and allowed for the reduction in the shock wave from the backing. This design approach improved the ballistic performance of ceramic armor systems. In addition, the thickness of the front ceramic plate could be reduced. A similar approach may be applied in the FGCA, utilizing one of the AM processing routes; in this case, a ceramic or metallic layer also filled with coarser grits may be produced.

For the FG-based components, specifically for FGCA, the entire manufacturing process and each processing step should be accompanied by related process control, as well as certification, as proposed in Figure 4. The detailed material and component design analysis, including the understanding of processing-structure-properties-performance, which should also include non-destructive testing and fractography upon ballistic testing, is highly important.

**Figure 18:**
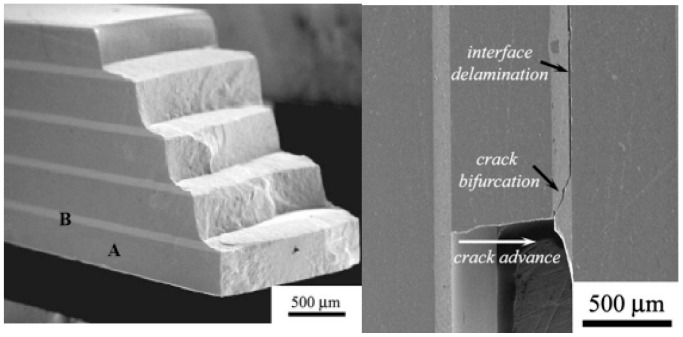
SEM Micrographs with the Laminate’s Step-Like Fracture and Crack Propagation under Flexure. Left—laminate’s step-like fracture associated with the compressive layers hindering the straight crack propagation; Right—crack propagation of a laminate under flexure, where a bifurcating crack approaches the interface at the layers A and B and causes interface delamination, while the structure underneath remains intact (acquired from [120]).

Basically, one of the major benefits of FGM is the creation of the specially designed layered structure of selected compositions, which delays crack propagation due to micro-crack deflection upon the applied mechanical loads, including impact [27,38,118,119]. The appropriate selection of the laminate architecture and composition of certain layers will allow for modifying the composites’ toughening mechanism, fracture, and crack propagation, and, overall, the possible enhancement of their ballistic performance. If the laminated composites have weak interfaces, it would be expected that the occurring delamination cracks and cracks’ deflection can promote energy dissipation during ballistic impact and fracturing. If the laminated composites have strong interfaces, it would be expected that the occurring cracks can deflect due to the residual stresses within the compressive layers. The propagating cracks’ re-orientation and deflection and/or bifurcation would lead to fracturing in stages, transforming from straight to step-like fracture (layer-by-layer). This type of crack propagation may be similar to that illustrated in Figure 18 [120]. According to Sun et al. [105], the interface SiC–BN layer (with ~1:1 ratio) would facilitate the crack kinking and delamination in the interface at fracturing, creating the step-like crack propagation. A similar effect was observed by Hong et al. [107], who studied the ballistic performance of laminated SiC-based ballistic ceramics where the interface layer promoted an energy absorption ability. Due to different mechanical properties of the matrix layers and the interface layer, the stress wave in the laminated composites would be reflected by the interface with following attenuation [18,31,33,109]. Thus, Li et al. [109] showed improved impact resistance of laminated ceramics due to multi-reflection and attenuation of the stress wave at the interfaces.

According to Bernejo and Danzer [120], who studied the fracturing of laminated composites, including those with various layers’ compositions, an optimal design, which promotes the crack bifurcation mechanisms followed by interface delamination, depends on a few factors. They include the following: (i) the level of compressive stresses defined by the layer thickness ratio and differential strain between layers; (ii) the combination of the layer thickness and compressive stresses, which causes crack bifurcation; (iii) the inclination angle of bifurcated cracks and elastic mismatch between the layers. Elastic properties of the constituents (e.g., moduli of elasticity, Poisson’s ratios) define the fracture energy in the composites. The residual thermal stresses between the layers with different compositions, which may occur during manufacturing, and related micro-defects should also be taken into consideration. This is a rather complicated design, and, to realize it properly, microscopy and fractography examinations of the materials under impact and finite element analysis may be employed.

## 5. Conclusions and Recommendations

The presented comprehensive review analyzes, for the first time, the possibility and potential of ceramic armor components, specifically armor with graded compositions and structures, using the AM approach. It includes a brief analysis of the positive and negative points of AM for the FGCA and considers its possible applications, comparing this technology with “conventional” commercial manufacturing of ceramic armor components. Since the armor design and technology may have numerous options, depending on specific situations and applications, they should be considered and selected in each particular case. The presented paper provides an important explanation and some guidelines, which can be utilized in the ceramic armor design, development, and manufacturing.

As a final remark, the AM technologies, specifically certain paths, like 3D- and direct ink printing, laminated object manufacturing with following infiltration, and some others, may create a unique possibility to produce lower-weight FG armor from different types of ceramics with complex shapes and various sizes for special designs. Moreover, considering AM technologies for ceramic armor systems, these technologies are the most reasonable for FG ceramic armor. In the FG armor design, the front layer with higher hardness, which will increase the projectile’s erosion and disintegration, and the layer(s) behind with higher fracture toughness can be recommended. The considered AM options can also be suited to reinforce the ceramic structures using selected ingredients, like platelets, fibers, meshes, rods, etc. Infiltration of the AM-based porous preform can be considered as a highly promising path providing bonding and consolidation of the ceramic structure. Reaction bonding can provide a new secondary phase formation, which should promote not only consolidation within individual ceramic layers, but also promote additional bonding of the layers, minimizing structural delamination, especially under ballistic impacts. The created laminated structures consisting of dissimilar materials should provide elevated toughness of the ceramic components, delaying crack propagation and possible localization of the energy of the shock waves under ballistic impacts. These structural features can promote ballistic performance, especially in multiple-hit situations. The proposed approach should assist in the creation of new ceramic armor designs and novel manufacturing capabilities, specifically for the armor systems with complex shapes and enhanced ballistic performance.

Despite the fact that significant progress has been made in the development of certain AM methods and in the fabrication of related AM processing equipment, as well as in the preparation of necessary ceramic-based starting materials, the biggest hurdles for the AM technologies are still the limitations imposed by the choice of materials for each AM method and the design considerations regarding the geometry of the part. Any future advances in AM must, therefore, be based on improving the existing technologies or developing new approaches, with a focus, in many cases, on the near-net-shape part forming. Manufacturing productivity and cost are highly important for the implementation of the components (ceramic armor) with FG-based designs and for related AM technologies. Emphasis should also be focused on the development of new materials or on the proper combinations of selected materials in FGM and optimal part design with minimization of the internal defects between the layers of dissimilar materials. Further work in these directions needs to be conducted, specifically focusing on the ceramic and composite compositions and industrially feasible processing routes of the complex shape armor components; they should provide a high consolidation level and homogeneity within the layers. In addition, ballistic testing and evaluation of the obtained FG armor systems need to be conducted. This is especially important since ballistic impact and related ceramic disintegration are significantly more complicated compared to these issues under slow (static) mechanical loading. Specifically, the speed of shock wave propagation and the crack propagation should be analyzed depending on the compositional and structural features of FGCA. The AM processing quality control, specifically focusing on the ceramics’ structural consistency, should also be critical in the consideration and implementation of this technology for ballistic applications. The applications may include personnel, vehicular, aircraft, and structural ballistic protection, mostly for specific designs and conditions.

## Figures and Tables

**Table 1:** Examples of Review Articles on Additive Manufacturing of Ceramic-Based Materials.

Authors	Year	Major Focus of the Review
J.D. Cawley [58]	1999	General review
J.W. Halloran [59]	1999	EFF, BJ, LOM, SLA
B.Y. Tay et al. [60]	2003	General review
J. Lewis et al. [61]	2006	EFF, BJ, DIP
X. Tian et al. [62]	2011	3DP, SLS, SLA, LOM, LSD
B. Qian and Z. Shen [63]	2013	SLS
N. Travitzky et al. [57]	2014	BJ, SLS, EFF, SLA, LOM
J. Deckers et al. [64]	2014	SLS, SLM, SLA
B. Derby [65]	2015	MJ
A. Zocca et al. [66]	2015	General review
J.W. Halloran [67]	2016	SLA
S.L. Sing et al. [68]	2017	SLS, SLM
L. Cheng et al. [69]	2018	General review
T.D. Ngo et al. [70]	2018	General review
B. Dermeik and N. Travitzky [71]	2020	LOM
J.S. Pelz et al. [72]	2021	General review
Y. Lakhdar et al. [73]	2021	General review
D. Grossin et al. [74]	2021	SLS
S.A. Rasaki et al. [75]	2021	SLA
S. Kirihara [76]	2021	SLA
J. Sun et al. [77]	2023	General review
M. Dadkhah et al. [78]	2023	General review
S. Bose et al. [79]	2024	General review
T. C. Dzogbewu et al. [80]	2025	SLS, SLM

**Table 2:** Properties of Some Studied Alumina Armor Ceramics (according to [3,13]).

Property	AZ	AL98	AL98.5	AL99.7	AM2
Density, g.cm^−3^ *	4.35–4.39	3.78–3.82	3.81–3.84	3.90–3.91	3.52–3.56
Young’s Modulus, GPa	325	325–360	370–420	400–450	235–242
Sonic Velocity, km.s^−1^	9.9	10.0–10.5	10.6–11.3	10.7–11.6	8.6–8.75
Vickers Hardness *HV10*, kgf.mm^−2^ (GPA)	1560 (15.6)	1220–1330 (12.2–13.3)	1320–1420 (13.2–14.2)	1520–1560 (15.2–15.6)	1130 (11.3)
Fracture Toughness *K_Ic_*, MPa.m^0.5^	4.0	3.2–3.3	3.3–3.4	3.1–3.4	2.5–2.55
Flexural Strength, MPa	530	250–350	270–360	320–380	350
Brittleness, *B* × 10^−6^, m^−1^	300	370–430	420–460	525–545	410–450
Ballistic Energy Dissipation Criterion *D* × 10^−12^, s^−1^ (calculated)	1.17	1.50–1.60	1.80–1.95	2.20–2.40	1.3–1.4

* Water absorption below 0.02% after firing at a temperature below 1550 °C.

## Data Availability

The original contributions presented in this study are included in the article. Further inquiries can be directed to the corresponding author.
